# Intravascular lithotripsy vs. rotational atherectomy on coronary microcirculation: a retrospective multicenter propensity-matched multicenter study

**DOI:** 10.3389/fcvm.2025.1560743

**Published:** 2025-05-07

**Authors:** Xi Zhang, Peijian Wang, Chenguang Li, Tao Zhao, Jiaji He, Bin Liu, Qing Jin, Pin Gan, Jilei Zhang, Qiang Xue

**Affiliations:** ^1^ Department of Cardiology, Yan'an Hospital of Kunming City, Yan'an Hospital Affiliated to Kunming Medical University, Kunming, China; ^2^ Kunming Cardiovascular Interventional Imaging Institute, Yan'an Hospital of Kunming City, Yan'an Hospital Affiliated to Kunming Medical University, Kunming, China; ^3^ Yunnan Key Laboratory of Cardiovascular Disease, Yan'an Hospital of Kunming City, Yan'an Hospital Affiliated to Kunming Medical University, Kunming, China; ^4^ Department of Cardiology, Clinical Medical College and The First Affiliated Hospital of Chengdu Medical College, Chengdu, Sichuan, China; ^5^ Department of Cardiology, Zhongshan Hospital of Fudan University, Shanghai Institute of Cardiovascular Diseases, Shanghai, China; ^6^ Department of Cardiology, The Fifth Affiliated Hospital of Kunming Medical University, Gejiu People’s Hospital, Gejiu, Yunnan, China

**Keywords:** intravascular lithotripsy (IVL), rotational atherectomy (RA), coronary microcirculatory function (CMD), angiographic microvascular resistance (AMR), peri-procedural adverse events (PPAEs), multicenter study

## Abstract

**Background:**

Coronary microvascular dysfunction (CMD) predicts poor prognosis in patients with coronary artery disease (CAD). However, the impact of intravascular lithotripsy (IVL) on CMD remains unclear, and no studies have directly compared IVL and rotational atherectomy (RA) in the context of CMD.

**Objective:**

This study aimed to evaluate CMD, as indicated by angiographic microvascular resistance (AMR), in patients undergoing IVL- or RA-assisted PCI for heavily calcified coronary lesions.

**Methods:**

This multicenter retrospective cohort study enrolled patients underwent either RA- or IVL-assisted percutaneous coronary intervention (PCI) at three centers. Propensity score matching (1:2) was performed to control for potential bias. The primary outcomes included the post-PCI AMR values and CMD incidence. The secondary outcomes included peri-procedural adverse events (PPAEs).

**Results:**

A total of 377 patients were registered, and 210 propensity-matched patients (140 RA vs. 70 IVL) were analyzed. Pre-PCI AMR was similar between the groups (RA 1.24 ± 0.53 vs. IVL 1.28 ± 0.50, *p* = 0.615). Following PCI, AMR was significantly higher in the RA group compared to IVL (2.43 ± 0.35 vs. 2.26 ± 0.50, *p* = 0.015), while CMD incidence was comparable (RA 32.9% vs. IVL 27.1%, *p* = 0.398). In addition, the PPAEs rates were lower in the IVL group but the difference showed no statistical significance (27.9% vs. 17.1%, *p* = 0.088).

**Conclusions:**

IVL demonstrates less microvascular dysfunction compared to RA, as indicated by lower post-PCI AMR. These findings suggest that IVL may offer advantages in preserving coronary microvascular function across various clinical scenarios when both techniques are equally available and applicable, but further large-scale prospective studies are needed to verify these results.

## 1 Introduction

With the aging population and increasing prevalence of diabetes mellitus and chronic kidney disease, calcified coronary artery disease (CAD) has emerged as a significant challenge in interventional cardiology (1, 2).

Calcified plaques in coronary arteries obstruct balloon dilatation and effective stent delivery, leading to malposition, stent under expansion, and drug-eluting polymer coat degradation (2, 3). These complications compromise procedural success and result in an elevated risk of ischemic events, which can severely impact long-term prognosis (4, 5).

Several treatment modalities have been developed to address this challenge (6, 7). Rotational atherectomy (RA) utilizes a rapidly rotating burr to ablate and modify calcified plaques and has demonstrated superior acute luminal gain and more successful stent delivery compared to balloon angioplasty alone (2, 5). Calcification debris was reported to cause coronary microvascular dysfunction (CMD). This translates to poorer procedural outcomes and increases the risk of major adverse cardiovascular events (MACE) (8).

In contrast, intravascular lithotripsy is a balloon-based device equipped with lithotripsy emitters. IVL induces fractures in intraplaque calcium by emitting sonic waves that are evenly distributed across the balloon surface, thereby facilitating plaque modification (9–11). Single-arm studies investigating IVL have reported promising results, including the prospective, multicenter DISRUPT CAD phase I, II, and III trials. Furthermore, IVL offers several advantages, such as lower debris generation and vascular intimal injury during the procedure, as well as a low complication rate (10, 12). Notably, the calcific lesions treated with IVL were generally less severe and complex than those addressed by RA (13), which may partially account for the observed differences in microcirculatory outcomes.

In theory, these advantages minimize damage to the microcirculation. However, the effect of IVL on coronary microcirculation function remains unclear due to the lack of trials comparing IVL to RA in the context of CMD.

The microcirculatory system can be evaluated using several methods, but noninvasive techniques such as positron emission tomography (PET) and index of microcirculatory resistance (IMR) remain underutilized. Among invasive assessment tools, wire-based IMR and coronary flow reserve (CFR) have seen limited use due to low repeatability and inconvenience despite being considered the most reliable indicators of microcirculatory function (14–16). Previous research has established that invasive measurements are independently associated with adverse outcomes in various cardiovascular diseases (17–19). To address these challenges, angiographic microvascular resistance (AMR) has been developed as a calculation method based on computational flow and pressure dynamics, providing a promising alternative to wire-derived IMR. AMR has gained popularity due to its simplicity and consistency with wire-based IMR. Previous studies have demonstrated that AMR is a viable alternative for assessing coronary microcirculatory function (20–22).

This study aims to compare the impact of RA or IVL on coronary microcirculatory function indicated by AMR in patients with calcified coronary artery disease treated with PCI. This study is the first to evaluate coronary microcirculatory function in patients undergoing IVL and highlights the first comparison of coronary microcirculatory function between IVL therapy and RA.

## 2 Methods

### 2.1 Study design and population

This multicenter and retrospective study included consecutive patients from September 2022 to September 2024 from three centers, namely Yan'an Hospital of Kunming City, Zhongshan Hospital of Fudan University, and The First Affiliated Hospital of Chengdu Medical College, who underwent PCI facilitated either by RA or IVL referring to the guidelines. Severe calcification was defined as either ≥270-degree arc of calcium on intravascular imaging, angiographic evidence of severe calcification blocking device passage, or a non-compliant balloon expand. The exclusion criteria in the clinical settings were as follows: (1) underwent both RA and IVL; (2) unavailability of follow-up data; (3) acute myocardial infarction; (4) left main PCI; (5) in-stent restenosis; (6) stenting of both the main and side branch. The exclusion criteria for angiography were: (1) missing preoperative or postoperative contrast; (2) AMR analysis could not be performed due to the inability to detect vessel borders or poorly filled contrast, excessive overlap of stenotic segments, or severely tortuous lesions in the target vessel. All interventions were performed in accordance with the guidelines outlined in the Declaration of Helsinki. The requirement for informed consent was waived owing to the retrospective nature of the study.

### 2.2 Procedural techniques

Coronary angiography was conducted according to conventional and local standards. An IVL catheter (Shockwave Medical, Santa Clara, CA, USA) was equipped with an integrated balloon that housed multiple lithotripsy emitters generating sonic pressure waves. Following the standard procedure, the balloon catheter was sized 1:1 to the reference artery and advanced over a coronary guidewire to the target lesion using a mono-rail technique. The balloon was inflated to a low pressure [4 atmospheres (atm)] to ensure vessel wall contact while minimizing the risk of barotrauma. Up to 10 impulses were delivered (1 pulse per second for 10 s, for a total of 80 pulses). Subsequently, the balloon was further inflated to the nominal pressure (6 atm) and then deflated to restore blood flow. In cases with multiple lesions, each lesion was treated with a minimum of 20 pulses.

RA was performed using the Rotablator (Boston Scientific, Marlborough, MA, USA) as per standard of care. Intraoperative irrigation was performed with heparinized saline to minimize the incidence of decreased blood flow.

Pre- and post-dilatation were permitted at the discretion of the operator. All patients received dual antiplatelet therapy prior to PCI, consisting of aspirin in combination with either clopidogrel or ticagrelor, as well as intraprocedural heparin administration, in accordance with current clinical guidelines (23, 24). Peri-procedural adverse events (PPAEs), including coronary slow flow or no flow after the procedure, coronary dissection, burr entrapment, side branch occlusion, peripheral vascular complications, contrast-induced nephropathy, myocardial injury, procedure-related myocardial infarction (MI), and in-hospital death, were recorded. Coronary slow flow/no flow referred to instant thrombolysis in myocardial infarction (TIMI) flow grade <3 post-procedure, without visible thrombosis, dissection, or spasm. Procedure-related MI was defined as an elevation of cardiac troponin (cTn) levels >5 times the normal upper limit, accompanied by recurrent symptoms with or without new ST-segment changes. Increased cTn values in patients with normal baseline values or a rise in cTn values >20% of the baseline were regarded as myocardial injury (25).

### 2.3 AMR computation and CMD definition

AMR was assessed by two independent specialists who were blinded to the patients' clinical characteristics and details regarding prior RA surgeries and clinical outcomes. Discrepancies in evaluation were settled by an independent analysis by a third specialist, and consensus was reached through subsequent discussions.

**Figure 1:**
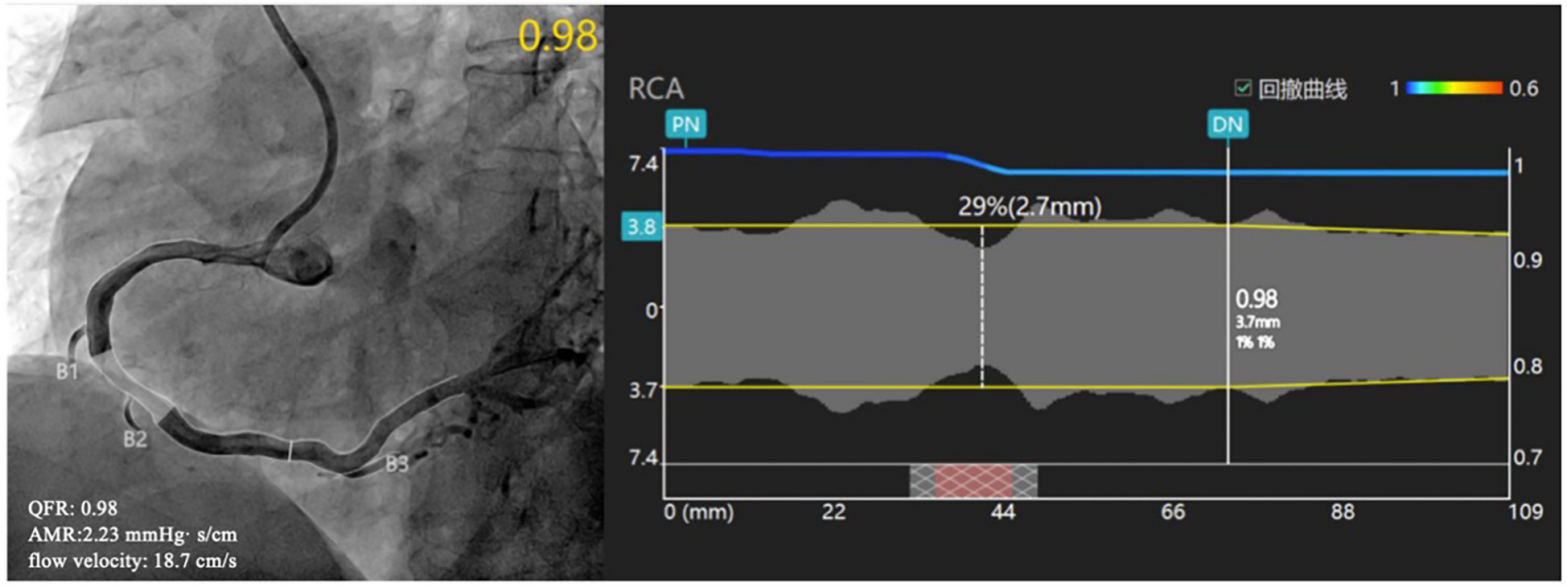
Examples of QFR and AMR analysis. CAG showed a stenosis in the right coronary artery, and QFR was calculated as 0.98, flow velocity was 18.7 cm/s, and AMR was calculated as 2.23 mmHg· s/cm. RA, rotational atherectomy; QFR, quantitative flow ratio; AMR, angiographic microvascular resistance; CAG, coronary angiography.

The methodology for calculating Murray's law-based quantitative flow ratio (*μ*QFR) and AMR has been previously described (Figure 1). Briefly, coronary angiography (CAG) images that met the predefined criteria were imported into AngioPlus software (Pulse Medical Technology). Coronary artery revascularization was conducted in accordance with Murray's law, and μQFR was calculated simultaneously with simulated flow velocities under hyperemic conditions (26). Distal coronary pressure (Pd) was derived from the pressure drop, and AMR was then calculated as the ratio of Pd to the simulated hyperemic flow velocity (Velocityhyp) (27).

$$\mathrm{AMR}=\frac{Pd}{\mathrm{Velocityhyp}}=\frac{Pa\times \mu \mathrm{QFR}}{\mathrm{Velocityhyp}}$$

The AMR measurement performed after CAG was defined as pre-AMR, and the measurement after PCI was defined as post-AMR. Patients exhibiting an AMR ≥2.5 mmHg-s/cm were categorized as having CMD (28).

### 2.4 Study endpoints

The primary endpoint was the comparison of the AMR value and rate of CMD between patients treated with IVL and RA. The secondary endpoint was the rate of PPAEs.

### 2.5 Statistical analysis

Continuous variables conforming to a normal distribution were expressed as mean ± standard deviation, while non-normally distributed data were expressed as median and interquartile range. Categorical data were presented as counts and proportions (%). Pearson's *χ*^2^ test and Fisher's exact test were performed for the comparison of categorical variables between groups, and the Mann–Whitney *U*-test was performed for continuous variables. A *p*-value of ≤0.05 was considered statistically significant.

Subsequently, propensity matching was performed for patients treated with IVL and patients treated with RA in a 1:2 ratio using the “Nearest Neighbor” method. The variables included in the propensity score were age, sex, smoking, diabetes, treated vessel, number of affected vessels, current dialysis, and left ventricular ejection fraction (LVEF).

Statistical analysis was carried out using IBM SPSS Statistics Version 26 (IBM, Armonk, New York, NY, USA) and R software version 3.6.3(R Foundation, Vienna, Austria).

## 3 Results

**Figure 2:**
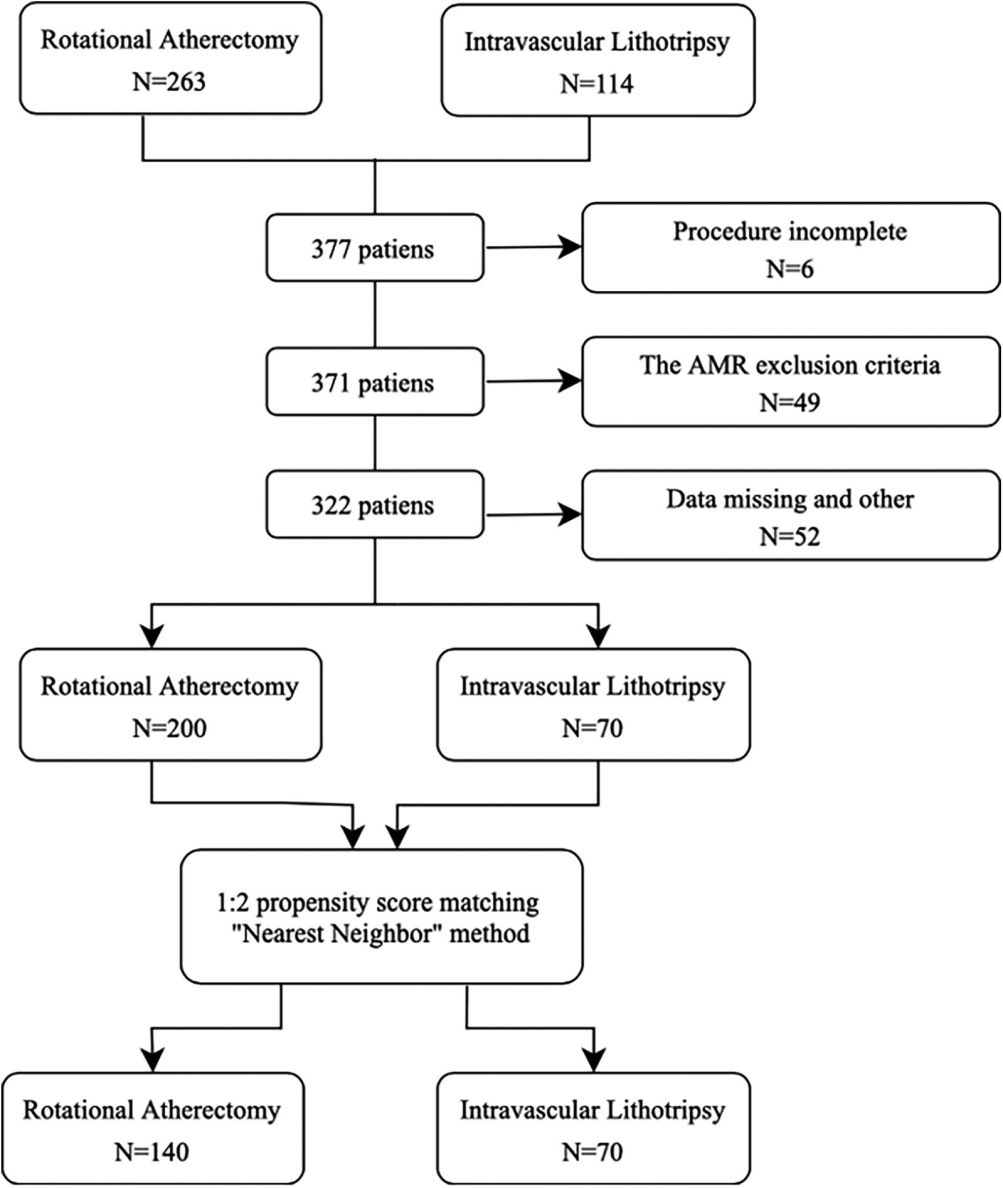
Flow chart of enrolled patients.

A total of 377 consecutive patients, of whom 114 underwent IVL and 263 RA, between September 2023 and November 2024 were enrolled in this study. The study flowchart is shown in Figure 2. After exclusions, 270 patients were considered for propensity score matching. The final population consisted of 210 patients, of which 70 were treated with IVL (53% male, median 65 years) and 140 patients with RA (58% male, median 66 years).

### 3.1 Baseline characteristics

As shown in Table 1, patients' clinical baseline characteristics, laboratory findings, and cardiovascular medical therapy among the two groups were similar. Both RA and IVL groups exhibited a high prevalence of comorbidities, including diabetes mellitus (44.3% vs. 38.6%), hypertension (62.1% vs. 67.0%), and current smoker (42.1% vs. 48.6%).

**Table 1:** Clinical baseline, angiographic, and procedural characteristics for the study population.

Variable	All patients	RA	IVL	*P*
	(*n* = 210)	(*n* = 140)	(*n* = 70)	value
Clinical baseline characteristics				
Age (years)	65.61 ± 8.59	65.95 ± 8.77	64.94 ± 8.23	0.800
Male, *n* (%)	118 (56.2%)	81 (57.9%)	37 (52.9%)	0.491
BMI (kg/m^2^)	24.43 ± 3.04	24.63 ± 2.79	24.02 ± 3.48	0.202
Hypertension, *n* (%)	134 (63.8%)	87 (62.1%)	47 (67.1%)	0.477
Diabetes mellitus, *n* (%)	89 (42.4%)	62 (44.3%)	27 (38.6%)	0.430
Dyslipidemia, *n* (%)	108 (51.4%)	69 (49.3%)	39 (55.7%)	0.380
TIA, *n* (%)	19 (9.0%)	12 (8.6%)	7 (10.0%)	0.734
CKD, *n* (%)	8 (3.8%)	5 (3.6%)	3 (4.3%)	0.799
Current smoker, *n* (%)	93 (44.3%)	59 (42.1%)	34 (48.6%)	0.377
Previous MI, *n* (%)	31 (14.8%)	19 (13.6%)	12 (17.1%)	0.492
Prior PCI, *n* (%)	143 (68.1%)	92 (65.7%)	51 (72.9%)	0.295
LVEF, *n* (%)				
≥50%	158 (75.2%)	104 (74.3%)	54 (77.1%)	0.902
35–50%	29 (13.8%)	20 (14.3%)	9 (12.9%)	
<35%	23 (11.0%)	16 (11.4%)	7 (10.0%)	
Laboratory findings				
HbA1c (%)	6.01 ± 0.94	6.04 ± 1.01	5.97 ± 0.81	0.627
LDL (mmol/L)	2.18 ± 0.75	2.17 ± 0.84	2.20 ± 0.53	0.771
HDL (mmol/L)	1.06 ± 0.27	1.06 ± 0.29	1.05 ± 0.23	0.720
GC (mmol/L)	2.28 ± 2.26	2.21 ± 2.25	2.41 ± 2.30	0.548
TC (mmol/L)	3.75 ± 0.98	3.80 ± 0.96	3.64 ± 1.03	0.268
Cr (μmol/L)	83.92 ± 70.47	82.81 ± 72.35	86.14 ± 67.00	0.748
Cardiovascular medical therapy				
Aspirin and its analogs, *n* (%)	210 (100.0%)	140 (100.0%)	70 (100.0%)	–
P2Y12 receptor antagonist, *n* (%)	210 (100.0%)	140 (100.0%)	70 (100.0%)	–
Statin, *n* (%)	203 (96.7%)	136 (97.1%)	67 (95.7%)	0.892
ACEI/ARB/ARNI, *n* (%)	161 (76.7%)	111 (79.3%)	50 (71.4%)	0.204
Beta blocker, *n* (%)	157 (74.8%)	108 (77.1%)	49 (70.0%)	0.261
CCB, *n* (%)	129(61.4%)	83(59.3%)	46(65.7%)	0.367

Values are mean ± standard deviation, median (interquartile range) or *n* (%); RA, rotational atherectomy; IVL, intravascular lithotripsy; BMI, body mass index; MI, myocardial infarction; PCI, percutaneous coronary intervention; CKD, chronic kidney disease; HbA1c, Hemoglobin A1c; LVEF, left ventricular ejection fraction; LDL, low-density lipoprotein; TC, total cholesterol; Cr, creatinine; ACEI/ARB, angiotensin-converting enzyme inhibitors/angiotensin receptor blockers; CCB, calcium channel blocker.

* *P* < 0.05.

Angiographic and procedural details are listed in Table 2. RA patients had a higher rate of stenosis in the target vascular region (88.6% vs. 85.0%) and post-dilatation (90.0% vs. 72.9%). In contrast, RA was associated with shorter stent length (33.57 ± 18.00 vs. 44.57 ± 15.49, *p* = 0.001) and lower frequency of lesions ≥20 mm (78.6% vs. 95.7%, *p* = 0.011).

**Table 2:** Angiographic and procedural characteristics for the propensity-matched population.

Variable	All patients	RA	IVL	*P*
	(*n* = 210)	(*n* = 140)	(*n* = 70)	value
Coronary physiological parameters				
pre-QFR	0.57 ± 0.18	0.57 ± 0.17	0.56 ± 0.18	0.856
pre-Velocityhyp (cm/s)	15.03 ± 4.75	15.15 ± 4.85	14.80 ± 4.59	0.622
pre-AMR (mmHg·s/cm)	1.26 ± 0.52	1.24 ± 0.53	1.28 ± 0.50	0.615
post-AMR (mmHg·s/cm)	2.37 ± 0.41	2.43 ± 0.35	2.26 ± 0.50	0.015*
△AMR (mmHg·s/cm)	1.11 ± 0.60	1.19 ± 0.61	0.98 ± 0.61	0.024*
CMD, *n* (%)	65 (31.0%)	46 (32.9%)	19 (27.1%)	0.398
Angiographic and procedural characteristics				
Target vessel, *n* (%)				
LAD	159 (75.7%)	104 (74.3%)	55 (78.6%)	0.715
LCX	16 (7.6%)	12 (8.6%)	4 (5.7%)	0.492
RCA	35 (16.7%)	24 (17.1%)	11 (15.7%)	0.295
Stenosis rate of target vascular area (%)	87.43 ± 6.71	88.65 ± 6.59	84.99 ± 6.30	0.0001*
Three-vessel coronary disease, n(%)	104 (49.5%)	71 (50.7%)	33 (47.1%)	0.626
Max Burr Size				
1.25 mm	–	29 (20.7%)	–	–
1.50 mm	–	87 (62.1%)	–	–
1.75 mm	–	19 (13.6%)	–	–
2.00 mm	–	5 (3.6%)	–	–
IVL Balloon Size (mm)	–	–	3.02 ± 0.29	
Postdilatation, *n* (%)	177 (84.3%)	126 (90.0%)	51 (72.9%)	0.001*
Number of stents	2 (2, 2)	2 (2, 2)	2 (2, 2)	0.117
Stent length (mm)	37.24 ± 17.93	33.57 ± 18.00	44.57 ± 15.49	0.001*
≥20 mm lesion, *n* (%)	177 (84.3%)	110 (62.1%)	67 (37.9%)	0.001*
Myocardial injury, *n* (%)	43 (20.5%)	34 (24.3%)	9 (12.9%)	0.053
Procedure related-MI, *n* (%)	18 (8.6%)	15 (10.7%)	3 (4.3%)	0.117
Peri-procedural adverse events, *n* (%)	51 (24.3%)	39 (27.9%)	12 (17.1%)	0.088
Instant TIMI flow grade <3, *n* (%)	13(6.2%)	11(7.9%)	2(2.9%)	0.265

Values are mean ± standard deviation, median (interquartile range) or n(%); RA, rotational atherectomy; IVL, intravascular lithotripsy; AMR, angiographic microvascular resistance; MI, myocardial infarction; PCI, percutaneous coronary intervention; Velocityhyp, simulated hyperemic flow velocity; CMD, AMR ≥2.5 mmHg- s/cm; △AMR = post-RA AMR—pre-RA AMR; LAD, left anterior descending artery; LCX, left circumflex; RCA, right coronary artery; TIMI, thrombolysis in myocardial infarction.

* *P* < 0.05.

### 3.2 AMR, myocardial injury, and peri-procedural adverse events

**Figure 3:**
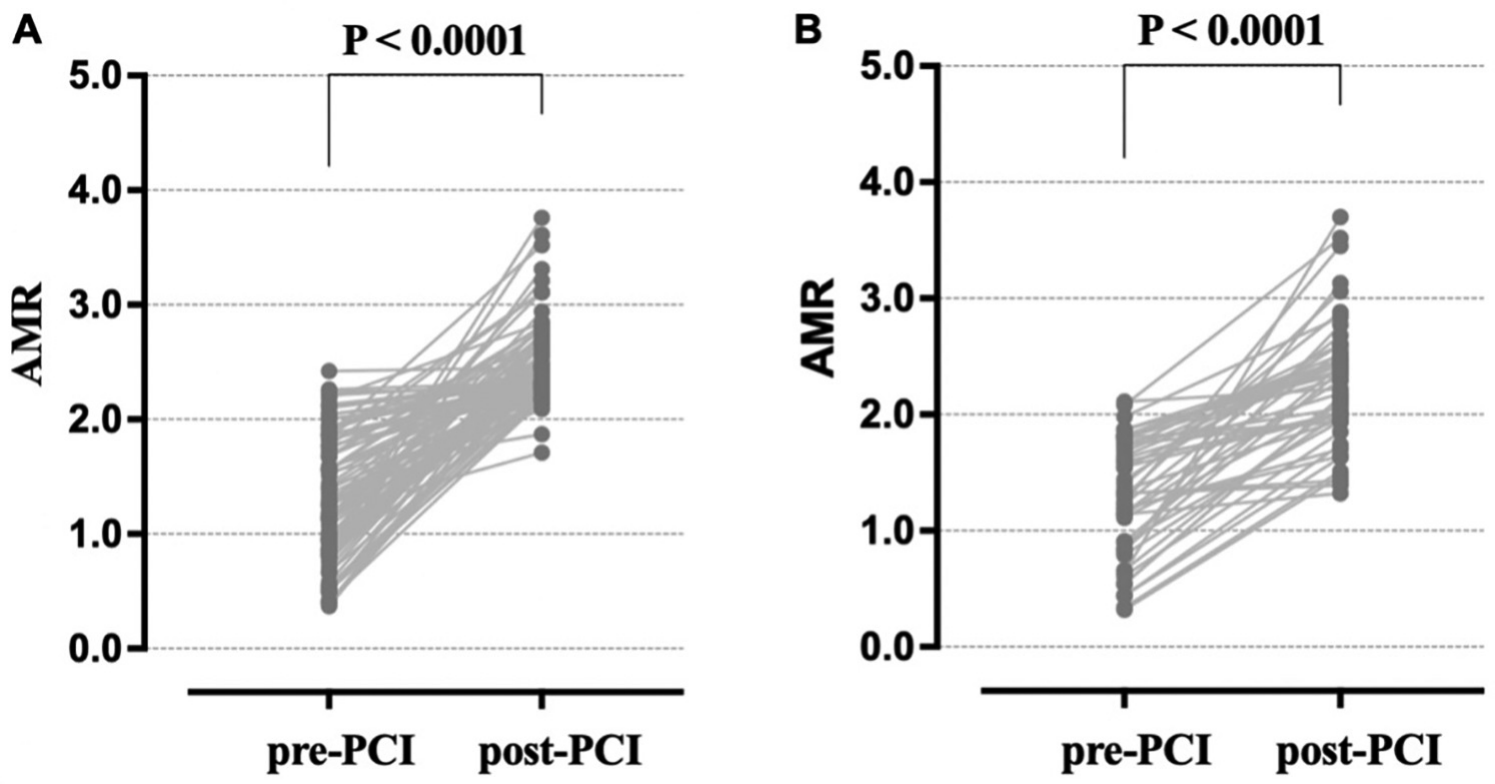
Temporal change of angiographic microvascular resistance (AMR) in the target vessels of the two groups. **(A)**: The AMR significantly increased in RA patients from pre- to post-PCI. **(B)**: The AMR significantly increased in post-PCI IVL patients compared to pre-PCI. AMR, Angiographic microvascular resistance; RA, rotational atherectomy; IVL, intravascular lithotripsy.

**Figure 4:**
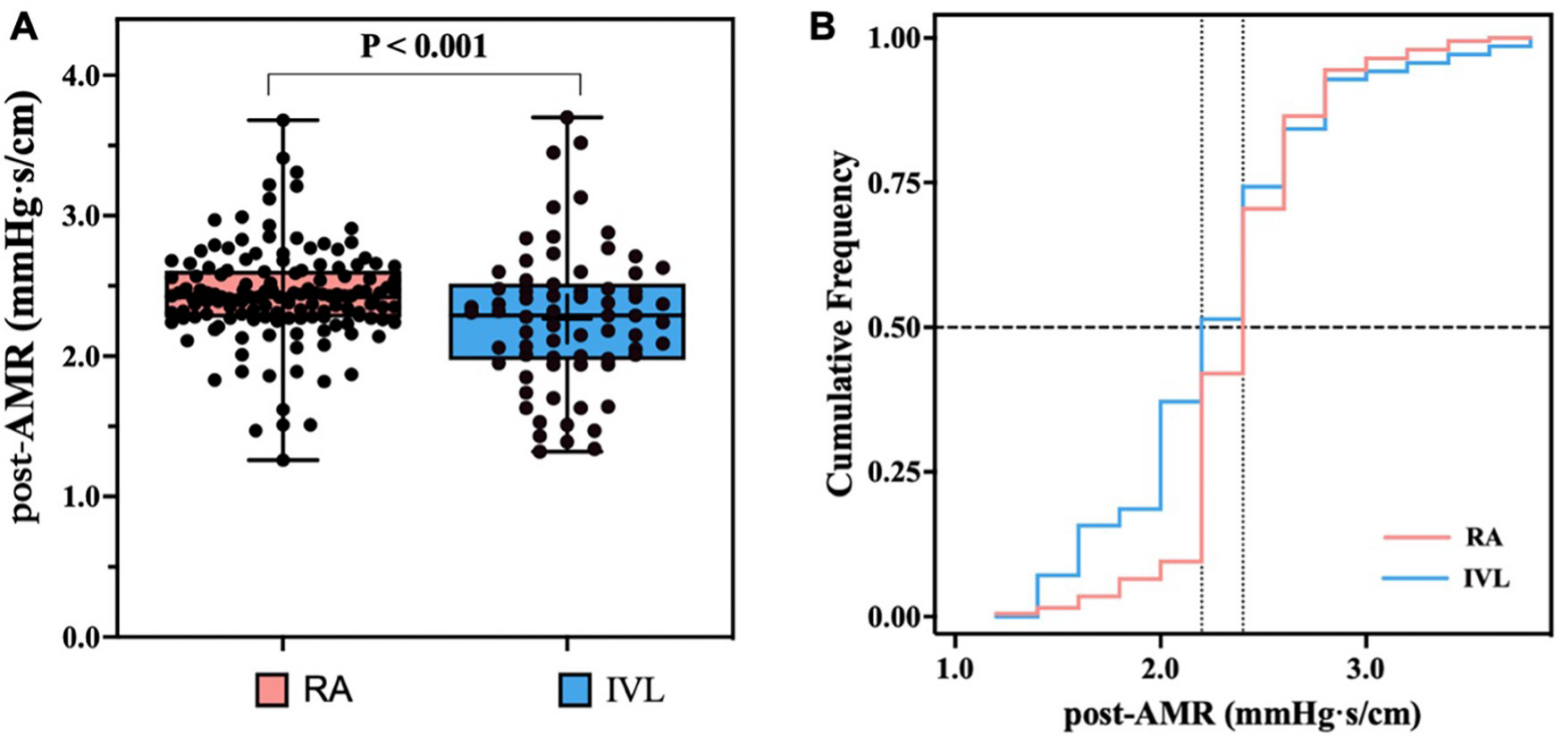
Angiographic microvascular resistance (AMR) after rotational atherectomy (RA) and intravascular lithotripsy (IVL). **(A)**: Box plot depicting the distribution of the post-PCI AMR between the two groups. **(B)**: Cumulative frequency distribution curves demonstrating lower AMR after IVL compared to RA. AMR, Angiographic microvascular resistance; RA, rotational atherectomy; IVL, intravascular lithotripsy; PCI percutaneous coronary intervention.

Table 2 illustrates the coronary microvascular function in the study population. Coronary microvascular function assessed by AMR was measured in 210 target coronary arteries, with the majority of lesions (75.7%) located in the left anterior descending coronary artery. Prior to intervention, similar AMR values were observed in the IVL and RA groups (1.28 ± 0.50 vs. 1.24 ± 0.53, *p* = 0.615), and no patients were diagnosed with CMD. Following PCI, AMR values increased in all patients (Table 2; Figure 3). However, the RA group showed a significantly greater increase in ΔAMR (1.19 ± 0.61 vs. 0.98 ± 0.61, *p* = 0.024) and post-AMR (2.43 ± 0.35 vs. 2.26 ± 0.50, *p* = 0.015) (Table 2; and Figure 4). After adjustment for procedure-related MI, the RA group still demonstrated significantly higher post-procedural AMR (adjusted *p* < 0.05) and *Δ*AMR (adjusted *p* < 0.05) compared to the IVL group.

A total of 65 patients (31.0%) developed CMD after procedure. Moreover, the incidence of CMD was comparable between the RA and IVL groups (32.9% vs. 27.1%, *p* = 0.398).

Additionally, IVL patients showed a lower incidence of PPAEs compared to RA patients (17.1% vs. 27.9%, *p* = 0.088), as well as fewer myocardial injury (12.9% vs. 24.3%, *p* = 0.053), procedure-related MI (4.3% vs. 10.7%, *p* = 0.117). A lower rate of TIMI flow < 3 was also observed, although this did not reach statistical significance (2.9% vs. 7.9%, *p* = 0.265).

## 4 Discussion

This study employed propensity-score matching to compare the impact of RA and IVL on coronary microcirculation function indicated by AMR. Our main findings are as follows: (1) the AMR value was significantly increased in all patients after PCI; (2) the post-PCI AMR value was significantly higher in patients who underwent RA compared to IVL; (3) the incidence of peri-procedural adverse events was comparable between the IVL and RA groups.

Interventional cardiologists now have an expanded array of tools to address severe coronary artery calcification, such as cutting balloons and lasers, but RA and IVL are the predominant techniques to treat calcified plaques (6, 29). While these interventions effectively relieve anatomic obstruction, the recovery of coronary microvascular dysfunction is crucial for improving patient outcomes.

In recent years, growing evidence has supported the use of CAG-derived IMR, which strongly correlates with wire-based IMR and has gained widespread adoption due to its simplicity (13, 28, 30). Notably, AMR, which does not require pressure guidewires or vasodilator drugs, has shown a robust correlation (*r* = 0.83, *p* < 0.001) and diagnostic performance (AUC 0.94; 95% CI: 0.91–0.97) when compared to wire-based IMR, using a single angiographic view (28). Previous studies have shown AMR's strong diagnostic ability in assessing CMD (AUC 87.2 95% CI: 83.0–91.3), demonstrating significant, independent associations with adverse events such as MACE and heart failure-related rehospitalizations (13, 20–22, 28, 31).

In our study, AMR values reflecting microcirculation dysfunction were significantly enhanced following PCI in all patients. Furthermore, recent studies have reported the effectiveness and safety of AMR as a tool for optimizing PCI outcomes (6, 32). Our results indicate that among patients with RA, the degree of CMD post-PCI was significantly higher than those treated with IVL.

Mechanistically, IVL distributes energy circumferentially, causing calcium fractures in multiple planes (30), which confers additional advantages such as reduced vascular intimal injury, preservation of vessel wall fibroelastic integrity, and a lower complication rate. Therefore, IVL induces lower microvascular damage compared to RA. On the other hand, RA pulverizes calcified lesions into fine particles that erode plaques (29). The size of these particles is smaller than that of red blood cells and can be efficiently cleared by the reticuloendothelial system (33, 34). Continuous active hypertension protection and a flushing solution (composed of adenosine, vasodilators, and heparin) were used in all RA patients, aiming to mitigate the impact of microthrombi and spasms.

The increased CMD observed following IVL was hypothesized to be primarily attributed to unavoidable atheromatous plaque debris, microvascular spasm, and embolization associated with the intervention (33, 35, 36). Nonetheless, this phenomenon may be reversible, warranting further investigation. Additionally, a greater volume of debris generated during the intervention is associated with more severe coronary microvascular dysfunction (37). RA in narrower and longer vascular lesions may cause an excessive amount of debris, potentially leading to system obstruction, myocardial injury, and subsequent distal microvascular dysfunction (33, 35). However, the unfavorable crossing profile of the IVL balloon remains a limitation. In contrast, RA can achieve superior acute luminal gain to easier cross in cases of very severe and balloon-uncrossable lesions, resulting in increased AMR (38). Generally, RA is mainly used for more severe and complex calcified lesions, which could explain the higher AMR and incidence of procedure-related MI in this group.

Notably, even after excluding patients with procedure-related MI—a known confounding factor for CMD and AMR elevation—the RA group still demonstrated significantly higher ΔAMR (*p* < 0.05) and post-AMR (*p* < 0.05) compared to IVL, consistent with the overall cohort results. These findings suggest that the microvascular protection advantage of IVL is independent of procedure-related MI occurrence.

Although RA patients showed a higher incidence of peri-procedural adverse events (27.9% vs. 17.1%, *p* = 0.088), including myocardial injury, procedure-related MI, and TIMI flow < 3, the small numbers and retrospective nature of this study precluded definitive conclusions. Larger prospective studies are warranted to validate whether the numerical trend reflects inherent differences between the techniques or is influenced by lesion selection bias (RA being used for more complex cases). Still, such negative clinical outcomes are likely to improve with further development of the technique and better case selection, enabling IVL treatment for complex calcified coronary lesions.

## 5 Limitations

The limitations of the present study should be acknowledged. First, the retrospective design inherently involves some bias, although measures such as propensity score matching and continuous enrollment were employed to minimize bias. Second, the potential confounding factors related to both patient and operator decisions could not be fully accounted for. Third, while the measurement of AMR was optimized using Murray's law and demonstrated high agreement with wire-based IMR, it remains less extensively validated than invasive physiological measurements. Fourth, adenosine was not routinely administered in this study to assess microcirculatory function, which limits our ability to differentiate between functional and structural coronary microvascular dysfunction. Finally, this study provided only a temporary assessment of microcirculatory function, and continuous monitoring was not conducted, which could offer more comprehensive insights into the dynamics of microvascular function. The role of AMR in optimizing the management and outcomes of microcirculatory dysfunction will be further studied in future research.

## 6 Conclusion

In patients presenting with calcified coronary lesions necessitating PCI, IVL resulted in lower post-AMR values compared to RA. Both groups exhibited similar rates of CMD (27% vs. 33%) and low rates of PPAEs (17% vs. 28%). Nevertheless, these preliminary results should be approached as hypothesis-generating. Substantiation of these findings necessitates larger-scale studies incorporating invasive physiological assessment, intravascular imaging, and clinical follow-up.

## Data Availability

The datasets presented in this article are not readily available because the data supporting the findings of this study are available from Yan'an Hospital of Kunming City, Zhongshan Hospital of Fudan University, and Clinical Medical College and The First Affiliated Hospital of Chengdu Medical College. Restrictions apply to the availability of these data which are used under license for the current study and are not publicly available. Requests to access the datasets should be directed to XueQiang, xueqiang3513@126.com.
